# Public Opinions and Concerns Regarding the Canadian Prime Minister’s Daily COVID-19 Briefing: Longitudinal Study of YouTube Comments Using Machine Learning Techniques

**DOI:** 10.2196/23957

**Published:** 2021-02-23

**Authors:** Chengda Zheng, Jia Xue, Yumin Sun, Tingshao Zhu

**Affiliations:** 1 Faculty of Information University of Toronto Toronto, ON Canada; 2 Factor Inwentash Faculty of Social Work University of Toronto Toronto, ON Canada; 3 Key Laboratory of Behavioral Science Institute of Psychology Chinese Academy of Sciences Beijing China; 4 Department of Psychology University of Chinese Academy of Sciences Beijing China

**Keywords:** Canada, PM Trudeau, YouTube, machine learning, big data, infodemiology, infodemic, public concerns, communication, concern, social media, video

## Abstract

**Background:**

During the COVID-19 pandemic in Canada, Prime Minister Justin Trudeau provided updates on the novel coronavirus and the government’s responses to the pandemic in his daily briefings from March 13 to May 22, 2020, delivered on the official Canadian Broadcasting Corporation (CBC) YouTube channel.

**Objective:**

The aim of this study was to examine comments on Canadian Prime Minister Trudeau’s COVID-19 daily briefings by YouTube users and track these comments to extract the changing dynamics of the opinions and concerns of the public over time.

**Methods:**

We used machine learning techniques to longitudinally analyze a total of 46,732 English YouTube comments that were retrieved from 57 videos of Prime Minister Trudeau’s COVID-19 daily briefings from March 13 to May 22, 2020. A natural language processing model, latent Dirichlet allocation, was used to choose salient topics among the sampled comments for each of the 57 videos. Thematic analysis was used to classify and summarize these salient topics into different prominent themes.

**Results:**

We found 11 prominent themes, including strict border measures, public responses to Prime Minister Trudeau’s policies, essential work and frontline workers, individuals’ financial challenges, rental and mortgage subsidies, quarantine, government financial aid for enterprises and individuals, personal protective equipment, Canada and China’s relationship, vaccines, and reopening.

**Conclusions:**

This study is the first to longitudinally investigate public discourse and concerns related to Prime Minister Trudeau’s daily COVID-19 briefings in Canada. This study contributes to establishing a real-time feedback loop between the public and public health officials on social media. Hearing and reacting to real concerns from the public can enhance trust between the government and the public to prepare for future health emergencies.

## Introduction

The World Health Organization declared COVID-19 to be a global public health emergency on March 11, 2020 [1]. Canada confirmed its first COVID-19 case on January 25, 2020, and a total of 189,387 cases of COVID-19 in Canada were reported as of October 14, 2020 [2]. On March 12, 2020, Quebec was the first province to declare a state of emergency [3], and the government established preventive measures such as transportation restrictions, quarantine rules, and social distancing requirements [4,5]. In Canada, Prime Minister Justin Trudeau started providing a daily briefing on the government’s updated policies and actions for stopping the spread of COVID-19 on March 13, 2020. For example, Prime Minister Trudeau announced an interest-free moratorium on student loan payments and closure of the United States–Canada border on March 18, 2020; announced the Canada Emergency Response Benefit (CERB) on March 25; and announced the Canada Emergency Student Benefit (CESB) through independent public entities (eg, lowering of the interest rate by the Bank of Canada) or federal government programs (eg, CERB and CESB) on April 22 [6].

Since the COVID-19 outbreak, social media has become the most accessible source for obtaining news and health information and for exchanging opinions. Given their self-isolation, people extensively post opinions, express emotions, and exchange ideas about COVID-19–related policies on the web [7]. In this study, we analyzed the YouTube comments under Prime Minister Trudeau’s daily briefing videos. YouTube is the first web-based video sharing platform. With more than 2 billion users and 1 billion hours of videos watched every day, YouTube has become one of the largest video sharing platforms worldwide, and it has played an essential role in public communication during the COVID-19 epidemic [8]. Comments on YouTube have provided rich data for public discourse and sentiment research. Existing studies examine the impact of YouTube videos and comments on epidemic diseases (eg, Ebola virus, Zika virus) and show that YouTube videos and comments are essential channels for disseminating health information [9-11]. Basch et al [9] coded the source and contents of the 100 most widely viewed videos about the Ebola virus on YouTube. They found that these videos have been viewed 73 million times and included contents about the death toll of the virus and how it was transmitted. Pathak et al [11] evaluated 198 YouTube videos about the Eloba outbreak in 2014. They found that most of the videos were useful, and they demonstrated that YouTube is a useful health information source during global health emergencies. In contrast, Bora et al [10] concluded that videos could potentially spread misinformation during global public health emergencies, and trustworthy videos from health organizations or universities were scarce. Khatri and colleagues [12] analyzed content about COVID-19 on YouTube by reviewing 72 videos in English and 42 videos in Mandarin; they demonstrated that YouTube is an important platform for health information dissemination. 

This study has two goals. The first is to examine whether YouTube comments are a useful source of public opinions and priorities on COVID-19; the second is to identify public responses and discourses related to Prime Minister Trudeau’s COVID-19 policies in Canada over time. According to Bora et al [10], we purposively selected trustworthy videos published by the official YouTube channel of the Canadian Broadcasting Corporation (CBC). More specifically, this study examines and tracks public discourse under Prime Minister Trudeau’s daily COVID-19 briefing videos posted on YouTube (N=57) from March 13 to May 25, 2020, in Canada. This paper contributes to understanding the undermeasured public responses to Prime Minister Trudeau’s COVID-19–related policies in Canada. Real-time and longitudinal analysis of public responses and concerns can help public health authorities recognize Canada’s public priorities.

## Methods

We followed the text mining pipeline, including data preparation and data analysis [7]. Data preparation included data sampling, collection, and preprocessing. Data analysis included unsupervised machine learning and thematic analysis. The unit of analysis was each unique comment posted under Prime Minister Trudeau's daily briefing videos from March 13 to May 25, 2020.

### Sampling

We used a purposive sampling approach. Our sampling frame was Prime Minister Trudeau’s COVID-19 daily briefing videos, which were published on the official CBC channel on YouTube [13] starting on March 13, 2020. Our sampling frame included 58 daily briefing videos posted from March 13 to May 22, 2020 (57 videos included comments; 1 did not receive any comments).

We present the characteristics of these videos in Multimedia Appendix 1, including publication dates; titles and descriptions of the videos; and numbers of views, likes, dislikes, and associated comments that each video received up to May 25, 2020. For example, on March 22, 2020, the daily briefing with the title “PM Trudeau announced that the $82-billion financial package was just the first step, and more financial funds will come” received the highest number of comments (n=2413) among the daily briefing videos published in March. The video titled “The term ‘New Normal’ to describe daily routine during the COVID-19 pandemic and the numbers would take months of continued, determined effort” had the highest number of comments (n=2087) in April. The video titled “Trudeau urged world leaders to pull together for the COVID-19 vaccine” received the highest number of comments (n=2653) in May.

### Data Collection

**Figure 1:**
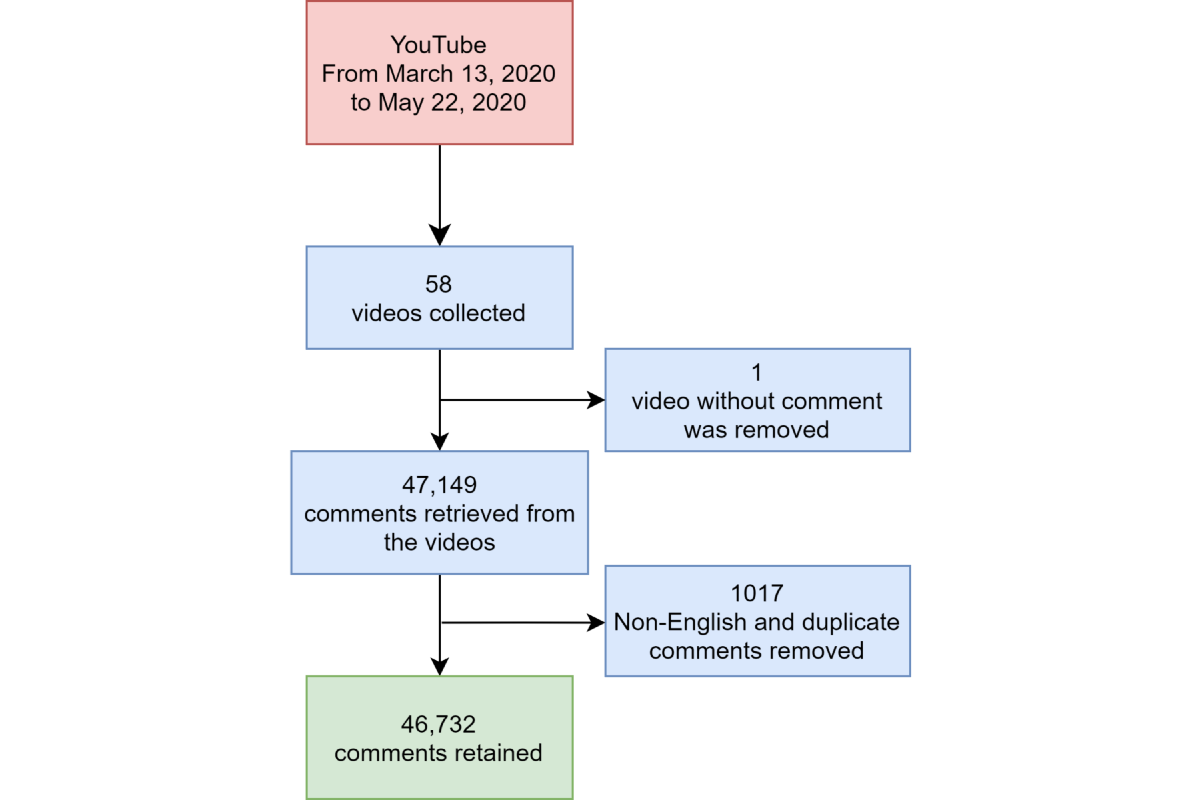
Data preprocessing chart.

The data set of the study consisted of all the comments posted by YouTube users under each of the 57 videos. We used YouTube’s open application programming interface to collect all the comments under each of Prime Minister Trudeau’s daily briefing videos on May 25, 2020. We coded the information retrieval script in Python, version 3 (Python Software Foundation). We retrieved a total of 47,149 comments from these 57 videos. After removing 1017 non-English and duplicate comments, 46,732 comments remained as our final data set, as shown in Figure 1. The following information associated with each of the 46,732 comments was collected: (1) a unique ID related to each comment; (2) the full comment text; (3) metadata associated with the comment, such as the reply count (number of replies that comment received), like count (number of the likes that comment received), published date and time of the comment, and deidentified username. The collected 46,732 English comments included both the first level comment and its associated “replyTo” (comment posted in reply to another comment) from 17,211 YouTube users.

We treated the set of comments published under each video as a separate document for the topic modeling (eg, documents 1-57). Thus, we obtained a total of 57 documents for topic modeling.

### Preprocessing of the Raw Data

We preprocessed the raw data to ensure data analysis quality. Preprocessing is an important task for analyzing YouTube comments, as it cleans the data by diminishing its complexity to provide better results [14]. Our data preprocessing included the steps below.

1. We removed handles (@user) and their content, as they did not contribute to the analysis. 
2. We removed all non-English characters (non–American Standard Code for Information Interchange [ASCII] characters) because this study focused on English messages.
3. We removed words that did not make sense, such as “#newYork sdaaawd asjdasd @!!!!” We used the Nostril evaluator [15] to detect nonsense comments and pronunciations. Nostril incorporates a large table of n-gram frequencies to support its probabilistic assessment of text strings. For the comments labeled as “nonsense” by Nostril, we also checked their pronunciations (ie, vowels and phones). The detected nonsense comments were removed from the analysis.
4. We removed stopwords to ensure better results [16]. Stopwords (eg, the, a, an, in) are frequently occurring words that do not carry any information or orientation. We used the Natural Language Toolkit suite of Python libraries to filter out the stopwords to save space in our database and shorten the processing time.
5. We applied a stemmer. A stemmer is a rule-based process of stripping the suffixes (eg, “ing,” “ly,” “es,” “s”) from words. A large number of conjugates were removed.

### Data Analysis

#### Unsupervised Machine Learning

We used latent Dirichlet allocation (LDA) [17] to generate prominent topics. LDA is a generative probabilistic model based on the hierarchical Bayesian that mines the underlying set of topics on a corpus of text. LDA has been applied to COVID-19–relevant topics on social media. For example, Xue et al [7] examined Twitter posts related to the COVID-19 pandemic. Obadimu and colleagues [18] applied LDA to recognize the toxicity of comments on YouTube. In this study, we treated the collected YouTube comments as a document and applied LDA topic modeling with the gensim Python library. For each identified topic, we used WordNetLemmatizer to extract popular unigrams and bigrams. The pyLDV library was used to visualize the findings.

#### Qualitative Analysis

The thematic analysis enabled us to interpret the patterns, topics, and themes from the unsupervised machine learning processes [19,20]. Two authors independently assigned topics based on the bigrams and representative comments related to each of the videos. Then, they reviewed all the identified topics and their representative unigrams, bigrams, and comment examples from the 57 videos and assigned themes across these topics. The research team examined the initial coded themes and considered whether they reflected the identified topics. The team discussed the themes and provided names to ensure that they reflected the identified salient topics.

## Results

### Descriptive Results of the Videos

Our sampling frame included 57 of Prime Minister Trudeau’s daily briefing videos published on the CBC YouTube channel between March 13 and May 22, 2020. We describe the statistics of the videos in Table 1, including the numbers of views, likes, dislikes, and comments of the 57 videos. For example, on average, these videos were viewed 364,487 times and received an average of 830 comments and 1826 likes.

**Table 1:** Descriptive statistics of the collected videos (N=57).

Characteristic	Range	Mean (SD)
Number of views	74,924-839,921	364,487 (205,984)
Likes	492-4405	1826 (1059)
Dislikes	290-2750	676 (344)
Comments	197-2653	830 (544)

**Figure 2:**
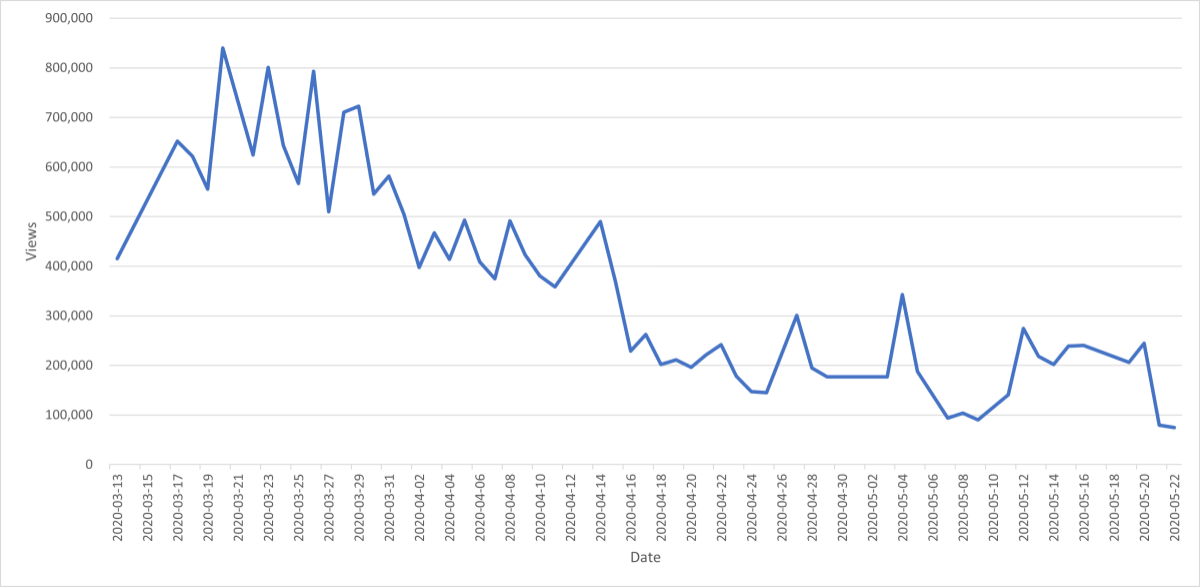
The number of views for the sampled daily briefing videos over time (n=56).

Figure 2 shows the view counts of Prime Minister Trudeau’s daily briefings over time. The view counts peaked on March 20 and gradually decreased after April, when Prime Minister Trudeau declared that the government would close the border to stop the spread of COVID-19 [21].

**Figure 3:**
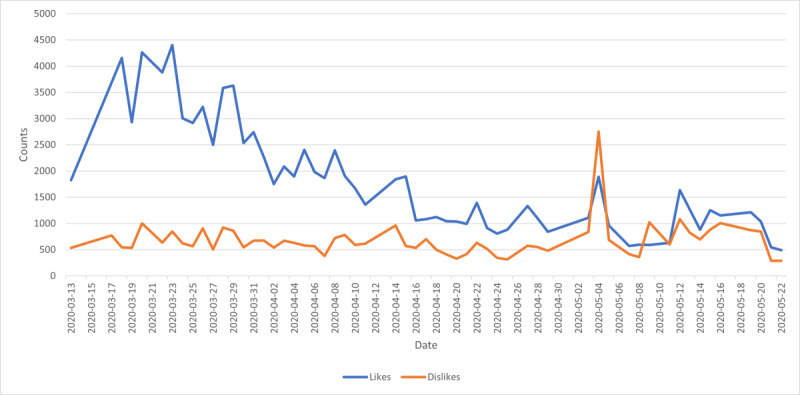
Like counts and dislike counts for the sampled daily briefing videos over time.

Figure 3 shows that the numbers of likes were higher than the numbers of dislikes on most of the days, except for May 4 and May 9, 2020. The daily briefing on May 4 urged all world leaders to work together and fight for a COVID-19 vaccine. Additionally, this video received the highest number of comments. Prime Minister Trudeau stated that Canadians would receive more financial benefits on May 9, 2020; this video received more dislikes than likes.

### Prominent Topics in the Comments

We retrieved and downloaded the associated 46,732 comments for each of the 57 videos as our final data set. We identified 1 to 3 salient topics for each of the documents (#1 to #57) associated with each daily briefing video, resulting in 168 topics. We present the topic modelling results for each of the 57 documents in Multimedia Appendix 2, including the publication dates of the videos, prominent topics in the documents, popular bigrams in the topics, and representative comments on each topic. For example, document #1 included all posted comments under Prime Minister Trudeau’s daily briefing on March 13, 2020. We identified two prominent topics in document 1: border measures and comment on the government. Popular bigrams were “close the,” “dam border,” “international travel,” and “prime minister.” A representative YouTube comment is “CLOSE THE BORDER!!!! Wake up Trudeau… close the god dam border Now. Stop all flights from Italy and Europe NOW.”

### Prominent Themes

Multimedia Appendix 3 shows the salient themes with their descriptions, topics (bigrams), and representative comments. We identified 11 themes from the 168 most prominent topics, including strict border measures, public response to Prime Minister Trudeau’s policies, essential work and frontline workers, individuals’ financial challenges, rental and mortgage subsidies, quarantine, government financial aid for enterprises and individuals, personal protective equipment, Canada and China’s relationship, vaccines, and reopening. For example, public response to Prime Minister Trudeau’s policies was a prominent theme, exemplified by the topic (uni)bigrams of government, Prime Minister, Justin Trudeau, Canada, officials, liberal, government and right wing. An example comment is “Respect and listen to the Prime Minister, he is trying his best with the Government to keep everyone in our Country safe from Covid19.”

**Figure 4:**
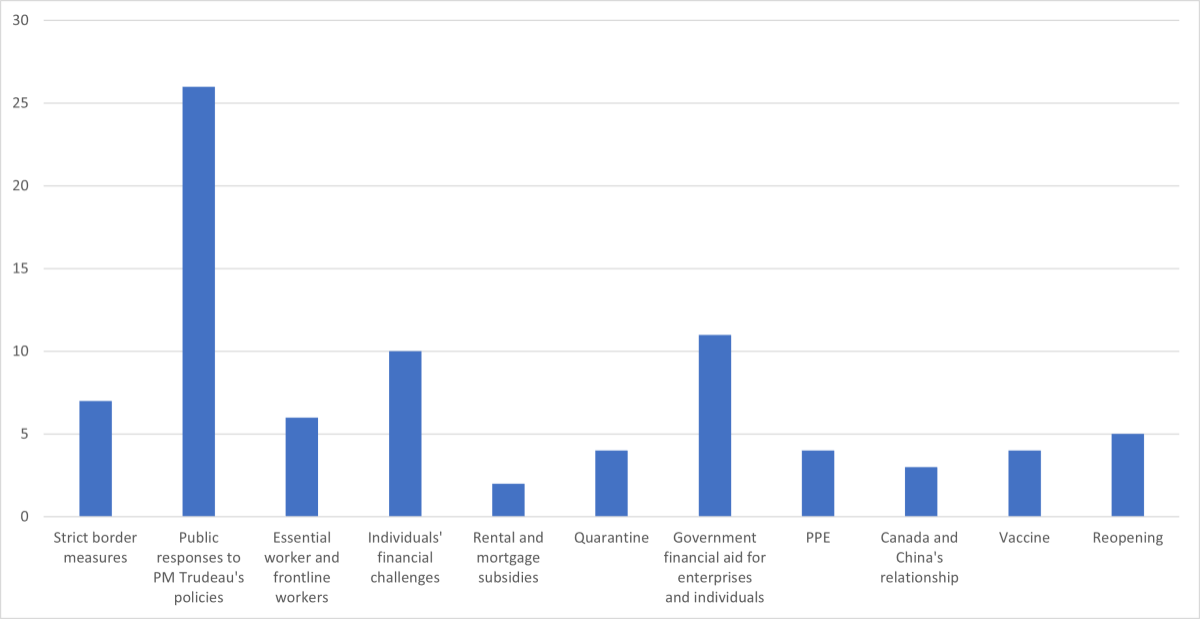
Occurrences of each theme from March 13 to May 22, 2020. PM: Prime Minister; PPE: personal protective equipment.

Figure 4 shows the number of occurrences of the prominent themes between March 13 and May 22, 2020. For example, the theme of public responses to Prime Minister Trudeau’s policies was the most popular, followed by government financial aid for enterprises and individuals and individuals’ financial challenges.

### Themes Over Time

**Figure 5:**
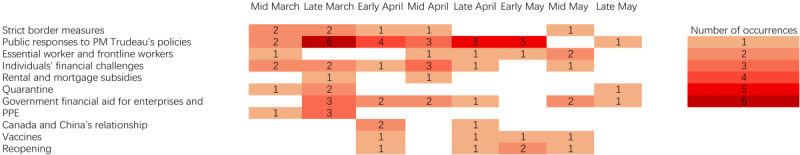
Prominence of each theme from March 13 to May 22, 2020. Early, day 1-10; mid-month, day 11-20; late, day 21 and after. PM: Prime Minister; PPE: personal protective equipment.

In addition to counting the number of occurrences, we also tracked the prominent themes over time, as shown in Figure 5. We divided each month into three periods: early month (day 1 to day 10), mid-month (day 11 to day 20), and late month (day 21 and after). Public concerns were ongoing for the themes of public responses to Prime Minister Trudeau’s policies and individuals’ financial challenges, as comments related to these themes appeared multiple times from March to May. For example, the theme of public responses to Prime Minister Trudeau’s policies had been continuously discussed by the public since March (except for in mid-May). The theme of strict border measures mainly occurred from mid-March to mid-April. However, it was discussed again in mid-May. The themes of vaccines and reopening were mainly discussed from late April.

### Popular Comments

We selected the 50 comments with the highest numbers of likes and the 50 comments with the highest numbers of replies. We present 19 comments that ranked in the top 50 most liked and replied in Table 2. The table shows the contents of these 19 comments along with their published dates, categorized themes, numbers of replies, and numbers of likes. For example, a single comment posted on March 28 received 488 likes and 104 replies from other YouTube users. This comment reflected the theme of strict border measures. Among the 19 selected popular comments, we found that five comments were about the theme of quarantine, and three comments reflected the theme of open government. The theme of quarantine was related to quarantine-related measures, as the public expressed a desire for more strict quarantine measures when they commented on and responded to Prime Minister Trudeau’s daily briefings. The theme of open government was related to the public’s request for a more open government and public interaction dynamics.

**Table 2:** Most popular comments, origins (daily briefing dates), assigned themes, and numbers of replies and likes.

Comment	Date of origin (2020)	Theme	Replies, n	Likes, n
“Why are we still not checking people at the airports?”	March 28	Strict border measures	104	488
“They should be pausing all mortgages and rent payments, the CRA can’t give you money by the 1st”	March 22	Rental and mortgage subsidies	99	525
“I really hope when this is defeated that our society doesn’t go more Orwellian”	May 04	Good wishes	88	230
“MR TRUDEAU: All you need to do is issue a moratorium stating that all Canadians with a rental lease or a mortgage is immediately suspended for 3 to 6 months. After which their existing monthly payments will restart and continue as usual. This will put at least 30% more money in every person’s account fairly. These funds can be used to pay cred it cards, car loans, food etc. It is a brilliant idea already being implemented in the USA by a number of Banks and institutions. This is simple and would help all Canadians. Obviously no penalty would by involved and this would be a Government approved legal postponement of all rents and mortgages for the period stated.”	March 24	Individuals’ financial challenges	78	246
“Its time to come together and support each other and question everything.”	May 04	Good wishes	68	802
“I don’t care about staying home. I’m a homebody anyways. I care about being poor and ending up completely homeless after the whole Covid19 crisis ends. My landlord already wants me to move out by August, and no one knows if it will have died down by then or if I’ll have a job by then so I can safely move.”	March 29	Individuals’ financial challenges	67	165
“Meanwhile, Canadian Tire, Lowes, Home Depot, Costco all the checkout attendants facing hundreds of customers per day and not one of them was wearing a mask and that was yesterday March 21st”	March 22	Quarantine	67	356
“Comments are allowed? Interesting”	March 26	Open government	66	335
“The scariest YouTube title ever”	April 09	Emotion	63	340
“I just want to know if these people are secretly getting haircuts. The rest of us will look like sheep dogs if this continues.”	March 26	Emotion	54	178
“I wish you didnt do the voice over, he is already doing it bilingually. we dont need to hear it two times in English besides the translation is badly done.”	April 14	Not related	54	615
“Anybody coming into Canada from other countries should have mandatory isolation for 14 days.”	March 28	Quarantine	53	222
“I dont understand why people still went on cruises even tho they were specifically told not to a longggggg time ago”	March 28	Emotion	51	269
“Lets have open questions from the public.”	April 09	Open government	40	203
“Close the border dont exempt American citizens. Close it down.”	March 17	Quarantine	38	304
“We should not rely on the US, like we are the 51st state!”	March 05	Emotion	34	180
“Dont have a social gettering of more than 10 people. Thats the guideline in South Korea. I think 50 is too much^^”	March 17	Quarantine	33	415
“Dr Tam waiting for ‘evidence’ of human to human transmission is disgraceful especially considering entire cities in China had already been locked down and the Three early German cases traced back to a Chinese business woman had already been reported. The precautionary principle should have been followed and Canadians deserve answers. We deserve better and Dr. Tam let us all down”	April 14	Quarantine	32	162
“Never in my life have I heard so many answers that dont answer a question. Ever. Lol”	April 14	Open government	32	317

## Discussion

### Principal Results

This study is the first to investigate and track public opinions and reactions to Canadian Prime Minister Trudeau’s daily COVID-19 briefings. This study uses a machine learning approach and longitudinally examines public discourse about Prime Minister Trudeau’s daily briefings over time. The results reveal several prominent themes over time, including strict border measures, public responses to Prime Minister Trudeau’s policies, individuals’ financial challenges, government financial aid for enterprises and individuals, and essential work and frontline workers. In addition, the themes of vaccines and reopening were highly discussed from late April. The study demonstrates that YouTube comments are a useful source of public opinions and priorities during global health emergencies.

#### Theme 1: Strict Border Measures

This theme refers to comments that discuss topics such as border entry policy, international travel, and border screening measures. The majority of the comments in this theme requested that the government implement stricter border measures to prevent transmission from carriers who have or do not have COVID-19 symptoms, exemplified by the comment “CLOSE THE BORDER... Stop all flights from Italy and Europe NOW.” Public discourse on this topic started in mid-March and remained a hot topic until mid-April. The results correspond with a policy announced by the Canadian government on March 18, 2020, that implemented a ban on all foreign nationals entering Canada, including restrictions on all nonessential travel at the Canada–United States border. Discussions of border measures were not prominent in late April and early May but reappeared in mid-May, consistent with the border closure agreement between Canada and the United States, which was valid until May 21.

Our results suggest that the “border policy issue” has continuously been a concern for Canadians, who support strict border measures to prevent the spread of COVID-19 in Canada. Previous studies demonstrated that air transportation has the potential to spread influenza, such as A/H1N1 [22], and enhanced precautions should be taken for travelers to countries with increasing cases of respiratory diseases, such as Middle East respiratory syndrome [23]. Brown and colleagues [24] showed that airport exit and entry screening at international borders facilitated the rapid detection of illness and the implementation of appropriate public health control measures to prevent the spread of Ebola virus. Our results suggest that future research can provide empirical evidence for the public and policy makers regarding the effectiveness of border measures to prevent the spread of COVID-19, as this has been a primary public concern over time.

#### Theme 2: Public Responses to Prime Minister Trudeau’s Policies

Public responses to Prime Minister Trudeau’s policies refer to public opinion comments on the Canadian government, parties, or the Prime Minister himself. The majority of comments include opinions regarding the government’s performance and policies during the COVID-19 pandemic. One representative comment is “Respect and listen the Prime Minister, he is trying his best with the Government to keep everyone in our Country safe from Covid19 with emergency funds for those that need it.” Compared to other themes, this theme is more popular, and it continuously received public attention from mid-March to late May. The popularity of this theme is due to the positive interaction dynamic between the general public and the government of Canada, which encourages people to communicate with government organizations on social networks. This interaction can be traced back to 2011, when Canada acknowledged the need to use social media to interact with the public for the first time [25]. Social media users in Canada are primarily interested in communicating with the government and obtaining customized information [26]. One study [27] showed that social media engages the public to foster participatory dialogues and discussions of policy development and implementation. In addition, we found criticisms related to the government’s COVID-19–relevant policies, such as:

Government Health Officials WHAT? They are months behind! Recommended NO MASKS only because they did not prepare, and they hadn't enough masks.

Previous studies have examined mixed sentiments toward governments’ policies in emergencies. During the 2009 H1N1 influenza pandemic, more than half the US population felt positive emotions toward the government’s policy responses, while a certain number of people did not [28].

#### Theme 3: Individuals’ Financial Challenges

Financial challenges refer to comments that include people’s discussion, concerns, or policy proposals related to financial loss during the pandemic, such as “having no income for essential living costs due to lost jobs.” One representative comment is:

I’m more worried about the financial issue than the health one right now. We are doing everything we can health-wise in this family, but my son is now out of work, and we know hubby will be soon.

Our results show that the public has been frequently and continuously discussing finance-related topics since Prime Minister Trudeau’s first daily briefing. The assessment of the impact of the COVID-19 pandemic on the labor market in Canada shows that the pandemic drove a 32% reduction in aggregate weekly hours worked from February to April 2020, along with a 15% reduction in employment [29]. As indicated by Statistics Canada, the average net savings for all Canadian households was CAD $852 (US $671) in 2018 [30], which suggests that people may be unable to afford essential living costs if they do not receive a basic income.

We found a positive correlation between the government’s policies supporting individuals and public attention to financial challenges. CERB was launched on April 6, 2020; nearly 3.5 million Canadians applied for this benefit in the first week, and this number increased to 7.12 million on April 24, 2020 [31]. More than 7 million people received CAD $2000 (US $1575) from the Canadian government in April, which explains the temporary decrease in the public attention toward financial challenges in early May.

#### Theme 4: Government Financial Aid for Enterprises and Individuals

Discussion about enterprises and individual financial aid plans was prominent from March to May 2020. This theme includes discussions of government financial aid plans and programs supporting either enterprises or individuals, such as CERB, CESB, the Canada Emergency Wage Subsidy, and the Canada Emergency Business Account. This theme is different from theme 3, individuals’ financial challenges, which focuses on individuals’ concerns and discussions of their financial obstacles during the outbreak. In late March, the theme of government financial aid became prominent when the government administration firstly announced a number of economic stimulation programs, exemplified by this comment: “If your business/source of income was interrupted due to COVID-19, whether you lost your business or not, you could still try to apply, as you would be in true need of help at this point.” Our results confirm those of previous research on emergency individual financial aid during the pandemic and the importance of financial aid programs for businesses [32,33]. The longitudinal discussions on financial aid plans suggest that financial aid during the COVID-19 pandemic is critical for the public, and further research is suggested to assess the need for financial aid programs and evaluate their implementation.

#### Theme 5: Essential Work and Frontline Workers

Essential work and frontline workers was a prominent theme in the comments related to Prime Minister Trudeau’s daily briefings, such as the welfare system in Canada, incentive pay for essential work, and protection of frontline workers. One representative comment stated, “The government should be incentivizing those who have to work during a pandemic, even a $500/month incentive.” This was an active public discussion topic in mid-March; it lost public attention for 20 days, and then became prominent again in mid-April. Our results are consistent with an earlier study that shows that frontline workers should be supported, and public support of frontline workers has been related to a speech by the Prime Minster of India [34]. Our findings suggest that Prime Minister Trudeau’s daily speech advocating for frontline workers also resulted in support and attention from the general public. For example, Prime Minister Trudeau mentioned essential work and frontline workers several times during his daily briefings, especially on April 29 and May 7. Our study suggests that political efforts in acknowledging essential workers has a substantial impact on the general public’s awareness. It is worth examining whether public acknowledgment influences people’s behaviors, such as strictly following social isolation and quarantine measures.

#### Theme 6: Vaccines

Public discussion of vaccines is another prominent theme that emerged in April. We classified all relevant topics in this theme, such as the development of vaccines, exemplified by this comment: “It could take years to come up with a vaccine, you’ve been doing a good job, Trudeau. Don’t it up by making statements you can’t back up.” Previous studies analyzing Tweets related to COVID-19 also found that vaccines were a highly discussed topic [7]. Our findings provide further evidence that vaccines have been a significant public concern among Canadians during the COVID-19 outbreak. However, we also found misinformation regarding vaccines on social media, where some comments supported that vaccines are a type of conspiracy. Our findings suggest that the Canadian government should provide more resources to the public to dispel misinformation regarding vaccines. Proper public education is essential to raise public awareness and to mobilize and engage people in communities in Canada.

### Limitations

There are several limitations to this study. First, this paper is not based on random sampling. We purposively collected comments that were posted by YouTube users under Prime Minister Trudeau’s briefing videos. They only represent a portion of the public opinions regarding Prime Minister Trudeau’s daily briefings during the COVID-19 pandemic, which causes biases. Second, our sample size is relatively small compared to the large amount of data on Twitter. Third, we treated secondary comments (ie, comment to comment) in the same manner as first-level comments for the study aims. Thus, this study has limitations in presenting the structural relationship between first-level and second-level comments. Finally, we retrieved all videos, comments, and associated data on May 25, 2020, three days after the last day of the Prime Minister’s daily briefing. It is noted that the evaluations could be different due to the increased number of comments added over time.

### Conclusion

This study is the first to analyze and track the public discourse of Canadian Prime Minister Justin Trudeau’s daily briefing on COVID-19 on social media. Machine learning analysis was applied to 46,732 English YouTube comments from 57 videos from March 13 to May 22, 2020, and the results suggest that several prominent themes should be noted by public health agencies and policy makers, such as strict border measures, public responses to Prime Minister Trudeau’s policies, individuals’ financial challenges, and government financial aid for enterprises and individuals. Recommendations for future work include (1) further validating YouTube comments as a source of information on COVID-19 and (2) strategies that can be considered by government and public health agencies to strengthen the real-time feedback loop between those agencies and the public on YouTube. Hearing and reacting to real concerns from the public can enhance trust between the government and the public to prepare for future health emergencies.

## Appendix

Multimedia Appendix 1 Descriptive characteristics of the sampling frame (N=57 videos).

Multimedia Appendix 2 Modeling of prominent topics for YouTube comments on each daily briefing (video dates, topics, bigrams, and representative examples).

Multimedia Appendix 3 Salient themes, topic keywords, and representative comments.

## Abbreviations

ASCII: American Standard Code for Information Interchange
CBC: Canadian Broadcasting Corporation
CERB: Canada Emergency Response Benefit
CESB: Canada Emergency Student Benefit
LDA: latent Dirichlet allocation
