# Supplementary material for: Public Opinions and Concerns Regarding the Canadian Prime Minister’s Daily COVID-19 Briefing: Longitudinal Study of YouTube Comments Using Machine Learning Techniques
Source: J Med Internet Res. 2021 Feb 23;23(2):e23957. doi: 10.2196/23957 (PMC7903980; doi:10.2196/23957)
Supplement: Multimedia Appendix 1 [file jmir_v23i2e23957_app1.docx]

**Appendix 1.** **Descriptive characteristics of the sampling frame (n=57 videos)**

| **Video Title** | **Publish date** | **Views** | **Likes** | **Dislikes** | **Comments*** |
| --- | --- | --- | --- | --- | --- |
| Trudeau calls on provinces to request COVID-19 testing and contact tracing help | 2020-05-22 | 74,924 | 492 | 292 | 267 |
| Trudeau pledges $75M in COVID-19 funding for Indigenous people living off-reserve | 2020-05-21 | 79,366 | 548 | 290 | 197 |
| COVID-19 update: Trudeau announces commercial rent assistance program | 2020-05-20 | 244,239 | 1,040 | 847 | 658 |
| Canada-U.S. border to remain closed for another month as provinces slowly reopen | 2020-05-19 | 206,306 | 1,214 | 872 | 996 |
| Trudeau acknowledges extreme impact of COVID-19 on Canada's travel industries \| Special coverage | 2020-05-16 | 240,578 | 1,154 | 1,010 | 808 |
| Liberals extend emergency COVID-19 wage subsidy program | 2020-05-15 | 238,822 | 1,252 | 881 | 713 |
| National parks, historic sites to partly reopen on June 1 | 2020-05-14 | 202,187 | 884 | 697 | 518 |
| Students can apply for COVID-19 emergency aid starting Friday | 2020-05-13 | 218,561 | 1266 | 824 | 788 |
| Seniors to receive up to $500 in one-time payment to offset COVID-19 costs | 2020-05-12 | 274,327 | 1,638 | 1,081 | 1,363 |
| Trudeau announces bridge loans for big businesses hit by COVID-19 | 2020-05-11 | 141,028 | 631 | 601 | 332 |
| Trudeau pledges more aid as public debt grows | 2020-05-09 | 90,119 | 592 | 1,025 | 321 |
| Trudeau says emergency wage subsidy will be extended | 2020-05-08 | 103,666 | 596 | 360 | 215 |
| Trudeau says $4B deal reached with provinces and territories to boost essential workers' pay | 2020-05-07 | 93,586 | 573 | 417 | 205 |
| Trudeau announces $252M emergency aid package for farmers, food processors hit by COVID-19 | 2020-05-05 | 187,771 | 961 | 685 | 647 |
| Trudeau urges world leaders to pull together for COVID-19 vaccine | 2020-05-04 | 342,772 | 1,891 | 2,750 | 2,653 |
| Trudeau announces $240M for online health-care services | 2020-05-03 | 177,252 | 1,110 | 838 | 535 |
| Trudeau stresses workers' safety at meat-processing plants | 2020-04-29 | 177,318 | 842 | 482 | 281 |
| New national data models show slower spread of COVID-19 | 2020-04-28 | 194,604 | 1,098 | 550 | 420 |
| Wage subsidy program opens, first payments expected May 7 | 2020-04-27 | 300,955 | 1,333 | 577 | 521 |
| Trudeau pledges $62.5M for fish and seafood sector \| Special coverage | 2020-04-25 | 145,196 | 882 | 314 | 405 |
| Trudeau offers rent relief for businesses affected by COVID-19 | 2020-04-24 | 147,125 | 808 | 343 | 405 |
| Trudeau earmarks $1.1B to COVID-19 science, as Sask. plans to reopen | 2020-04-23 | 178,100 | 911 | 515 | 508 |
| Students to get $9B in aid, Trudeau announces | 2020-04-22 | 241,416 | 1,396 | 633 | 697 |
| Students to get $9B in aid, Trudeau announces | 2020-04-22 | 241,416 | 1,396 | 633 | 697 |
| Trudeau pledges $350M to community groups helping seniors, homeless | 2020-04-21 | 221,071 | 994 | 415 | 427 |
| Trudeau addresses Canadians on Nova Scotia mass shooting | 2020-04-20 | 196,522 | 1,037 | 328 | 560 |
| Federal parties grapple over reconvening Parliament | 2020-04-19 | 211,264 | 1,042 | 408 | 450 |
| Canada, U.S. agree to extend border restrictions, Trudeau says | 2020-04-18 | 202,000 | 1,124 | 500 | 444 |
| Trudeau earmarks $1.7B to clean orphan wells and expands business credits | 2020-04-17 | 262,702 | 1,082 | 701 | 574 |
| Trudeau plans to expand aid for businesses, assistance for commercial rent | 2020-04-16 | 228,847 | 1,056 | 538 | 358 |
| Part-time, contract and seasonal workers now qualify for CERB \| Special coverage | 2020-04-15 | 368,561 | 1,895 | 570 | 718 |
| Trudeau announces new mandatory COVID-19 measures \| Special coverage | 2020-04-14 | 489,673 | 1,842 | 961 | 1,517 |
| Trudeau, opposition leaders and select MPs debate wage subsidy bill \| Special coverage | 2020-04-11 | 358,301 | 1,360 | 614 | 509 |
| Trudeau highlights interest-free loans to help small businesses during COVID-19 | 2020-04-10 | 380,689 | 1,672 | 590 | 749 |
| 'This is the new normal,' until COVID-19 vaccine developed: Trudeau | 2020-04-09 | 423,051 | 1,909 | 783 | 2,087 |
| COVID-19 update: Trudeau says more help coming for youth, businesses | 2020-04-08 | 491,475 | 2,394 | 722 | 610 |
| Canada working to produce up to 30,000¬†ventilators: Trudeau | 2020-04-07 | 374,757 | 1,867 | 380 | 531 |
| COVID-19 update: Trudeau says benefits could be deposited within 3 to 5 days of applying | 2020-04-05 | 492,528 | 2,405 | 580 | 1,055 |
| Trudeau promises more Canadians to receive COVID-19 emergency benefit | 2020-04-06 | 408,953 | 1,982 | 567 | 961 |
| COVID-19 update: Trudeau says 'millions of masks' coming from China \| Special coverage | 2020-04-04 | 413,646 | 1,898 | 631 | 1,128 |
| COVID-19 update: Trudeau recalls Parliament to pass enhanced emergency aid | 2020-04-01 | 504,533 | 2,268 | 675 | 794 |
| Trudeau announces $100M in food bank funding \| Special coverage | 2020-04-03 | 467,118 | 2,088 | 673 | 781 |
| Trudeau to discuss co-ordinated COVID-19 strategy with premiers \| Special coverage | 2020-04-02 | 397,629 | 1,753 | 539 | 631 |
| Trudeau says made-in-Canada medical supplies coming soon \| Special coverage | 2020-03-31 | 581,919 | 2,738 | 673 | 972 |
| COVID-19: Trudeau gives details on wage subsidies, loan programs for businesses \| Special coverage | 2020-03-30 | 545,453 | 2,535 | 544 | 1,144 |
| COVID-19 update: Trudeau pledges more help for vulnerable Canadians \| Special coverage | 2020-03-29 | 722,372 | 3,628 | 863 | 1,698 |
| COVID-19 update: Trudeau limits domestic travel for symptomatic Canadians | 2020-03-28 | 710,702 | 3,583 | 924 | 1,587 |
| COVID-19 update: Trudeau announces measures to help small businesses | 2020-03-27 | 509,566 | 2,497 | 508 | 447 |
| COVID-19 update: Trudeau implements Quarantine Act | 2020-03-26 | 792,682 | 3,222 | 906 | 1,661 |
| COVID-19 update: Goverment passes $107B aid package for Canadians | 2020-03-25 | 566,581 | 2,914 | 567 | 1,212 |
| COVID-19 update: MPs set to vote on emergency aid for Canadians | 2020-03-24 | 643,490 | 3,009 | 623 | 1,187 |
| COVID-19 update: Trudeau warns of potential enforcement measures | 2020-03-23 | 800,700 | 4,405 | 846 | 1,481 |
| More financial aid to come, Trudeau says | 2020-03-22 | 624,225 | 3,882 | 637 | 2,413 |
| COVID-19: Canada to turn back asylum seekers, says Trudeau \| Special coverage | 2020-03-20 | 839,921 | 4,263 | 1,002 | 0 |
| COVID-19: Trudeau says closures, social distancing possible for weeks or months | 2020-03-19 | 555,583 | 2,932 | 536 | 982 |
| COVID-19: Canada unveils $82B emergency response package \| Special coverage | 2020-03-18 | 621,741 | 4,158 | 548 | 652 |
| COVID-19 update: Trudeau addresses Canadians \| Special coverage | 2020-03-17 | 652,212 | 3,687 | 771 | 1,325 |
| Avoid international travel to stop spread of COVID-19, officials say \| Special coverage | 2020-03-13 | 415,098 | 1,827 | 535 | 354 |
| *All metadata were retrieved and collected on May 23, 2020. | | | | | |
