# Supplementary material for: Public Opinions and Concerns Regarding the Canadian Prime Minister’s Daily COVID-19 Briefing: Longitudinal Study of YouTube Comments Using Machine Learning Techniques
Source: J Med Internet Res. 2021 Feb 23;23(2):e23957. doi: 10.2196/23957 (PMC7903980; doi:10.2196/23957)
Supplement: Multimedia Appendix 2 [file jmir_v23i2e23957_app2.docx]

**Modeling prominent topics for each daily briefings YouTube comments (video dates, topics, bigrams and representative examples**

| **Dates** | **Prominent topics** | **Bigrams** | **Representative Example** |
| --- | --- | --- | --- |
| 3/13 | 1. Border measures 2. Comment on the government | 1. close the, dam border, international travel, avoid,  2. government, prime minister, should be | 1. CLOSE THE BORDER!!!!!!!!!! Wake up Trudeau... close the god dam border Now. Stop all flight from Italy and Europe NOW. 2. Trudeau and RCMP should be held responsible for infecting Canadian Citizens during this virus epidemic. |
| 3/17 | 1. Stay at home, 2. Essential work | 1. stay home, to protect, distancing, 2. work, people need | 1. These people either choose to work or choose to stay home and be safe. 2. i volunteer at a local food bank in Halifax, we have to stay open because hungry people need to eat. |
| 3/18 | 1. Border measures, 2. Individual financial problems | 1. border right, the border, people cross, 2. payments, have work, bill | 1. the problem cant be solved when we continue to allow flights and people to cross our borders daily. Especially with next to no screening even admitted by noncanadians in the airports. 2. sold my car for the down payment, Im on quarantine but I still need to keep the lights on, we have property tax, my utility bill is around 300 a month, groceries 300 … |
| 3/19 | 1. Comment government, 2. Individual financial problems | 1. government, trudeau is, Canada, 2. paid, what they, basic income | 1. stop giving free healthcare that Canadian taxes pay for to people coming here to take advantage of our weak government and laws while canadians suffer without. 2. Most people live paycheque to paycheque, what are they supposed to do when their work has shut down due to government order and are not being paid. |
| 3/22 | 1. Individual financial problems 2. Rent, landlord | 1. financial, paycheck, bill, 2. landlord, mortgage, tenant | 1. I think for anybody who work and get a regular paycheck why do they need to get money because they work if this can happen, they are not falling on any kind of hardship. 2. No penalties so landlords with mortgages are put on a pause, so that they can offer up to three months rent help for their tenants. |
| 3/23 | 1.Quarantine  2. Comment on the government | 1. social distancing, essential, quarantine 2. government, Trudeau is, Canada | 1. Temperature should be checked at any essential facilities like grocery stores, I see sick people buying food. they should be given help to get it delivered. 2. Trudeau you said Canadians fine covid19 just a cold you ignored the fact such as closed down the aiports and boarders and now your telling us to obey you because its worst then you thought the reason why its worst because you let it happened |
| 3/24 | 1.Comment government, 2. financial problems | 1. Trudeau, Canada, the government, 2. tax payers, help, money | 1. They are a sly and manipulative liberal government Thanks to the opposition for stopping this over reach of power. 2. Can someone send me a time stamp where he explains the plan is help Canadians with money and support |
| 3/25 | 1.Governments financial aid plan | 1. government, interrupted, people who | 1. In general they would have to prove it yes, as this addresses those who have been interrupted from their normal income because 1) they got sick and ordered to stay home 2) caring for someone who is sick with covid-19 and other related issues. |
| 3/26 | 1. Border measures 2. Comment government | 1. the border, our country, virus, 2. Canada, the government, prime minister | 1. Trudeau was allowing migrants into our country from the States and no media has criticized him for this…how many of these people brought the virus…Canadians not closing the illegal crossing sooner. 2. mismanaging a pandemic response can be catastrophic, so lets count our blessings that our government is utilizing data driven decisions and focus some positive energy on those who need it right now - those working the frontline of this pandemic. |
| 3/27 | 1. Commen government 2. Government financial aid plan | 1. government, Canadian 2. government, income, businesses, financial, | 1. Thank you to our public health providers, researchers for doing all they can to avert the worst health scenarios; all levels of government working together to address, help 30+million family emergencies;  2. $5 billion a week the government is spending to keep big businesses open how about the government give that to the people and watch our economy grow we need houses we can start small businesses we can spend the money put it back into our country our money |
| 3/28 | 1. Quarantine  2. Border measures | 1. virus, social distancing stay home, isolation,  2. Canada, the airport, world, screening | 1. Government should require every one healthy or not, to wear face mask, gloves and wash hands always so that we can prevent the virus to spread from one person to the other and stay home. 2. What is there flights still coming in and no one being screened. Canadians are do their part so it time Trudeau do the part. |
| 3/29 | 1. Comment government | 1. government, Canadian, Trudeau | 1. I praise Trudeau for all he has done so far and I am so glad I am Canadian after seeing what is going on in the U.S. So glad his wife and kids are now safe and healthy, and I hope they stay that way. |
| 3/30 | 1. Industry relief fund | 1. employment, government, employee | 1. Will this program allow currently working employees to be paid while having their hours cut to 75% then use entirely government money to pay their employees and take 100% profits after material costs. |
| 3/31 | 1. Comment government, 2. Medical equipment | 1. Canada, government, Trudeau, 2. Production, canada medical, equipment, health care | 1. Respect and listen the PM, he trying his best with the Government to keep everyone in our Country safe from Covid19 with emergency funds for those that need it. 2. Im glad Prime minister Trudeau all supplies needed for COVID 19 to help the health care worker is going to be made in Canada. Please try not to accept anything coming from China yet. Thank you very much and please Stay safe. |
| 4/1 | 1. Comment government | 1. Canada government, Canadian, prime minister | 1. Humanity Is Awakening, Rise Over Tredeau Corrupt Government They Evil Corrupt Government controlling Canada brought the virus into the country let it spread freely, now they are days away from putting martial law against Us! |
| 4/2 | 1. Medical equipment 2. Canada &China | 1. equipment, medical supplies, protective 2. China, tonnes, aircraft carrier | 1. I think it is more logical take every precaution not to get sick, rather than sending my small personal supplies to the hospital! I am also troubled that officials are forgetting about essential services, postal workers, social workers, personal care nurses, police...who need medical grade protective supplies, masks, rubbing alcohol to make wipes, and so on. It is disappointing that our health officials discourage public masks for small reasons, and either dont recognize that protective personal supplies add to public protection, as they did in S Korea, China, Singapore, New York, Italy. 2. Well Trudeau shipped sixteen tons of our medical equipment over to China over the course of five days in Early February, when 5 to 7 Canadians had already been infected with COVID-19. |
| 4/3 | 1. Food bank | 1. Trudeau, food bank, immigrant | 1. there needs to be regulations that are food banks because all the Muslim immigrant refugees that Trudeau allowed to come into our country are very verbally and aggressively abusive when it comes to food bank they go in there and they take everything and they dont leave nothing for anybody. |
| 4/4 | 1. Canada &China 2. Medical equipment | 1. from china, masks from, china when 2. mask, medication, own masks, factories | 1. All the countries need to stop depending so much on products from China. million masks just sent were mostly donated from Huawei the china company Canada has treated so poorly and Huawei was still so kind as to help Canada medical staff when our own PM is not. 2. Agreed we should produce mask here in Canada most important need which each country should produce not to depend on others. |
| 4/5 | 1. Medical equipment 2. Financial problems | 1.companies, government, manufacturing, medical supply, 2. benefit, apply for, income | 1. The steps will have to be taken to stockpile the necessary medical supplies each country needs to navigate through an entire pandemic. It will require a global logistics and supply chain that can only operate with the cooperation. 2. If my income in 1 year no more than 5.000 even i work with the same job almost 5 month but only get very little hours do im eligible to get cerb benefit. |
| 4/6 | 1. qualification of CERB 2. Border measure | 1. people who, apply for, the money, qualify for, 2. international, come to Canada, international student | 1. They are going to have a whole bunch of people with no income apply to this benefit hoping to qualify because they really need the help and confused if they do or not. 2. Right, how the Airport still open flights are people coming in Canada, we needed stop all immigrations, visitors, students from the other countries,same Asian, but the government doesn’t care the risks from Covid19 in Canada country in future again ? |
| 4/7 | 1. Comment government | 1. Canadian, Trudeau, justin trudeau | 1. Mr. Trudeau sounded sincere and real concerned for Canadians unlike our TV reality president who wanted to impress his base supporters and turned his daily WH briefing into a political rally, and for those who know the facts and truth. |
| 4/8 | 1. CERB qualification  2. Comment government | 1.financial, apply for, the money, 2. Canadian, Trudeau | 1. OTTAWA – The government has a warning for those who unduly apply for both Employment Insurance and the new Canada Emergency Response Benefit (CERB): we will find you, and we will recoup the money. 2. People relax please and help Trudeau. Canadian tax payer would save a lot of money if Trudeau would be asked to stay at his cottage and never be allowed in the parliament. |
| 4/9 | 1. Vaccine | 1.a vaccine, bill gates, immune system | 1. Bill Gates believes the world should only have 300 million people and he is the one beginning human trials on his vaccine. |
| 4/10 | 1. Re-open | 1. small business, essential, quarantine, distance | 1. I have been closed for 30+ days, and my Fixed expenses are around 20k/month....my family will pay for this, and that’s not possible...I guess it was a good run. if grocery stores can operate with people milling about and a hundred people in line outside for weeks without becoming major hot zones. there must be a safe way, with proper protocols in place, to open smaller businesses and get more people working and save some businesses. |
| 4/11 | 1. Comment on the government 2. Financial problems | 1. Canadian, justin trudeau, provincial, care about, 2. wage subsidy, pandemic, debt relief, government | 1. As a senior citizen at the age of 72, I have been constantly criticizing the Trudeau’s Federal Government about why his economic relief plan does not include senior citizens and Disabled Canadians who rely on fixed pension income and disability assistance respectively. 2. Any comment that actually deals with the debt relief the government is wanting to offer Canadians to prevent them from bankruptcy in this time of pandemic? |
| 4/14 | 1. Trudeaus travel to cottage | 1. his cottage, his family, trudeau is, quarantine | 1. And people in Ontario are getting tickets for going out for a bike ride while our King (oops Mr True though - Trudeau) pulls his stunt with his family at the cottage country while snapping happy pics (yet we are told to celebrate our Easter in self isolation). |
| 4/15 | 1. CERB qualification | 1. last year, you qualify, apply for, thank you | 1. What about new immigrants who arrived in Canada in January (just before this virus spreaded), continuously applying for a job and not getting any response because of current market conditions? Are they eligible for CERB? Thank you. |
| 4/16 | 1. Comment on the government 2. Financial problems | 1. canadians, Canada, prime minster, Trudeau 2. difficult, financial, unable to | 1. Thank you Mr: prime minister Trudeau for helping Canadian people and trying to do your best for your country. 2. So.....financial aid for most but since I was unemployed before this started, I am being forced to find work and put myself at risk. 3 children, my wife had to stop working because of covid and if she gets CERB, I lose my ODSP. |
| 4/17 | 1. Federal oilpatch bailout | 1. government, energy sector, oil companies, liabilities | 1. Alberta has a responsibility, as PM Trudeau stated, to insure if oil fields created a mess, that mess be cleaned, such that FARMERS have a right access to THEIR arable land. |
| 4/18 | 1. Border measure 2. Financial problems | 1. the border, canadians, traveling, restrictions, 2. essential, the economy, money | 1. Trump advised POS Trudeau that the border will stay closed another 30 days. 2. We are international students came to Canada in January and we were supposed to work in April but due to lockdown we are unable to get to work so we dont have any money to pay our rents , grocery , phone bills and home utilities. |
| 4/19 | 1. Comment on the government 2. Essential work | 1. government, prime minister, politicians 2. essential, essential workers, their jobs | 1. Its time to drag Trudeau out of his safe space and get to the business of asking him the hard questions that he can never seem to answer. 2. They are essential workers, they should all go back to work, like I have to do each day. All the people working right now are lower/middle class with the exception of doctors but in Canada, I would still consider them middle class. |
| 4/20 | 1. Gun control | 1. gun control, nova scotia, government | 1.sadly the politicians will use this to try and take our guns away from the good law abiding citizens instead of focusing on the criminal aspect and illegal guns. |
| 4/21 | 1. Financial problems, 2. Comment on the government | 1. taxpayers money, please help, income, 2. thank, leadership, pm | 1. Same, usually I work at a factory during the summers to be able to afford going to school, but because of this virus, Im out of that job and my only source of income. 2. Thank you PM Trudeau for trying to help people during difficult times. Hope you become better and better in your job. |
| 4/22 | 1. CESB & Student Assistance 2. Comment on the government | 1. international, students, assistance, 2. government, Canadian, Trudeau | 1. not every student has the luxury to live at home rent free, plus many of us rely on summer jobs to pay for tuition and living expenses for the school year. 2. I couldnt take Trudeaus war on the Middle Class anymore so I now live in the U.S. I make more money, I am taxed less, and I am paid in a currency is actually worth something. |
| 4/23 | 1. Vaccine | 1. the virus, the vaccine, trudeau, bill gates | 1. The only question worth knowing Mr. Trudeau is when the heck are we gonna get a vaccine for the COVID? |
| 4/24 | 1. Rent and landlord 2. qualification of CERB | 1. landlords, mortgages, rent relief, 2. government, Canadian, million dollar, assistance | 1. Businesses need rent relief, but landlords have mortgages to pay, so the solution is to freeze mortgages so rents can be frozen too. you know how many people are going outside for the new sunny weather and not listening to your stay home advice Small business gets rent relief. 2. I hope the government of Canada would even simplify more the CERB requirements because, there are still some more Canadians who are in need of financial assistance badly for some reasons, and I am one of them. |
| 4/25 | 1. Financial aid for fish and seafood 2. Comment on the government | 1. and seafood, prime minister, 2. prime minister, government, trudeau | 1. Prime Minister Justin Trudeau announced $62.5 million for Canada fish and seafood sector amid mounting concerns over the state of the country food supply. 2. When we are having a difficult time from terrible Wuhan Coronavirus pandemic attacking like this that we could see how great full of our prime minister for taking good care of our country our citizens! |
| 4/27 | 1. re-open | 1.to reopen, government, economy | 1. I think there needs to be a little more thought put into any reopening of the economy. That scenario does not even consider the fact that a second wave of Covid 19 could come along and put us all in same situation we were in when economy came to screeching halt first time. |
| 4/28 | 1. Essential work 2. Comment on the government | 1. social distance, stay home, work, protect 2. government, Canadian | 1. Underpaid staff working without personal protective equipment and allowed to work in more than one nursing home in order to make ends meet, bringing in the virus and spreading it because they are taking care of so many different people and not washing their hands. 2. If a million Canadians had died from this virus, you people who are critizing our government for being cautious and enforcing physical distancing, would be crying that our government did not do enough to save people. |
| 4/29 | 1. Epoch times | 1. epoch times, the epoch | 1. For those who think Epoch Time a good source for news they will soon see it will work in unison with the US to remove any government in Canada who dares to disobey the wish of the US. |
| 5/3 | 1. Gun control 2. Comment on the government | 1. freedoms, assault rifle, gun, 2. government, Justin trudeau | 1. Very few Canadian guns migrate from legal to illegal and when you consider our low gun crime the evidence is right in front of you. 2. If you are sick and tired of Justin Trudeau coming out of his hole every morning gaslighting. Trudeau is taking your guns because he plans to do something so bad to you that you would need guns to defend yourself. Trudeau is rewriting the Charter of Rights and Freedoms without lifting a pen. Time to overthrow this gaslighting PRICK. |
| 5/4 | 1. vaccine | 1. vaccine, rest of the world, bill gates | 1. Dear rest of the World, there a lot of people in Canada that DO NOT support what this Prime Minister is doing: spending hard earned tax payers money in helping getting a vaccination - therefore endangering the health of many, but making the Pharma Industry richer then before, making Gates amp; more powerful, and with all this pushing the UN Agenda 2030! |
| 5/5 | 1. Comment on the government | 1. government, trudeau is | 1. Well one good lie that we dont know where its not a lie Justin Trudeau is from Pakistan so he has family has a lot of money in Pakistan so if he brings all his family here to Canada whos going to be in exchange for cash five years from now can you use will have to move out of this country because there’s no room for Canadians Trudeau has all his family here and Friends |
| 5/7 | 1. Comment on the government 2. Essential work | 1. trudeau, rightwing 2. workers, pay, essential, workers | 1. We dont need is money what we need is to remove Trudeau the traitor and treason him with is corrupted cabinet rich cabal elites and turning Canada into a muslims isis iran Islam country Trudeau for jail. 2. As an essential worker who has not stopped working since this thing happened ive so far received $0 from the government. |
| 5/8 | 1. Comment on the government 2. re-open | 1. government, Justin , trudeau, 2. workplaces, essential, haircuts | 1. The best part of The Morning Show with Justin is either how he can talk for so long without saying anything or the fact that if you listen closely, you can almost hear the wind blowing through his ears. 2. People need haircuts its one of the oldest job you got to adapt to time otherwise its bankruptcy and the state cant sustain this level of passive support. |
| 5/9 | 1. Comment on the government 2. re-open | 1. canadians, the country  2. essential, business, to reopen | 1. I used to be a Trudeau Fan but I am growing more and more disappointed with him skirting around NOT answering the tough questions...instead he evades them by regurgitating the same things he says everyday. 2. All of you people wanting businesses to reopen despite the danger of the still pandemic covid-19 are morons. This is basically for businesses like Pho Hoa or local pizza place, they need people to get the business going, but their income might be drastically lowered and cant support employment. |
| 5/11 | 1. re-open | 1. big business, small business, re-open | 1. Big business just need people to work and they will be fine. Small businesses need people to work, and they will be fine. |
| 5/12 | 1. frontline workers 2. Financial problems | 1. government, people on, what about  2. government, the money | 1. Canadian Government always remember giving additional money to seniors, jobless people, refugees, etc....but they have forgotten to give extra compensation to frontline healthcare workers specially nurses and care aides who have direct contact to patients....all the government does is praise frontline workers as heroes...but no extra compensation whatsoever but rather continues to deduct humongous amount of taxes! 2. You said it,,we should all be screaming as tax payers in the streets,,we work hard, the government takes our money and gives it away and screws us over every chance they get,,it is near impossible to get ahead in our country,,cut our federal income tax you take off our pay, cut our municipal house tax! |
| 5/13 | 1. CESB & Student Assistance 2. frontline workers | 1. application, international, student 2. government, work, during pandemic | 1. These direct aids will be misused by a large portion of students, just like the Liberals wasting our tax funds. 2. The government should be incentivizing those who have to work during a pandemic, even a $500/month incentive would help wonders, but yet theyre giving money to those who dont actually need it.The government should be incentivizing those who have to work during a pandemic, even a $500/month incentive would help wonders, but yet theyre giving money to those who dont actually need it. Its about time governments pay up to the working people in this country instead of rich corporations. |
| 5/14 | 1. vaccine | 1. vaccine, virus, bill gates, scientist | 1. Together we sew reap and fall,Let us work together to bring succes to stop this virus from taking over our world,by speeding up the vaccine to save lives. |
| 5/15 | 1. CERB penalties | 1. penalties, apply, cheque | 1. I work at money mart..Id say half the people cashing their CERB cheques should not be receiving them. |
| 5/16 | 1. Aircanada | 1. air canada, government, AC | 1. CBC: Stop reporting Air Canada 🇨🇦, we are all suffering, watching AC cry for bailing out make us SICK , they need to cope themselves, we already bail them before they misuse them to buy share back and pay CEO millions dollars |
| 5/19 | 1. Situation in U.S 2. Border measure | 1. U.S, Trump, america 2. the border, Canada, to protest | 1. i believe america is going to regret their decision , and i dont want to see canada making the same mistakes , we as canadians are better than this , we need to teach ourselves to just be patient. 2. The border needs to stay closed until either the US gets COVID under control or there is a strict testing regime at the point of departure for every person crossing the border (for x-border flights, before allowing the person on a plane). |
| 5/20 | 1. Comment on the government | 1. government, Canada, in Canada | 1. Thanks for supporting I Love Canad my home because govermant in Canadian responsible to people God bless you all |
| 5/21 | 1. China & Canada 2. qualification of CERB | 1. china social, china, credit system, 2. government, the money, financial | 1. ziman China recently acquired the rights to a newly-discovered gold deposit in Nunavut, up in the Canadian north. 2. In the meantime Im stuck on welfare because I dont qualifyt; Im considered 3 different answers when I called CERB help line but yet I worked last year and payed taxes. |
| 5/22 | 1. Tracing for live case | 1. contact tracing, government, virus | 1. Contract tracing is unconstitutional and illegal Id love to see anyone try and force me to stay anywhere even in my own home. you will never lock ppl up and take away their rights unconstitutionally and illegally without repercussions of some kind People are sick of daddy govt overreaching just go away. |
