# Supplementary figures and images for: Public Opinions and Concerns Regarding the Canadian Prime Minister’s Daily COVID-19 Briefing: Longitudinal Study of YouTube Comments Using Machine Learning Techniques
Source: J Med Internet Res. 2021 Feb 23;23(2):e23957. doi: 10.2196/23957 (PMC7903980; doi:10.2196/23957)

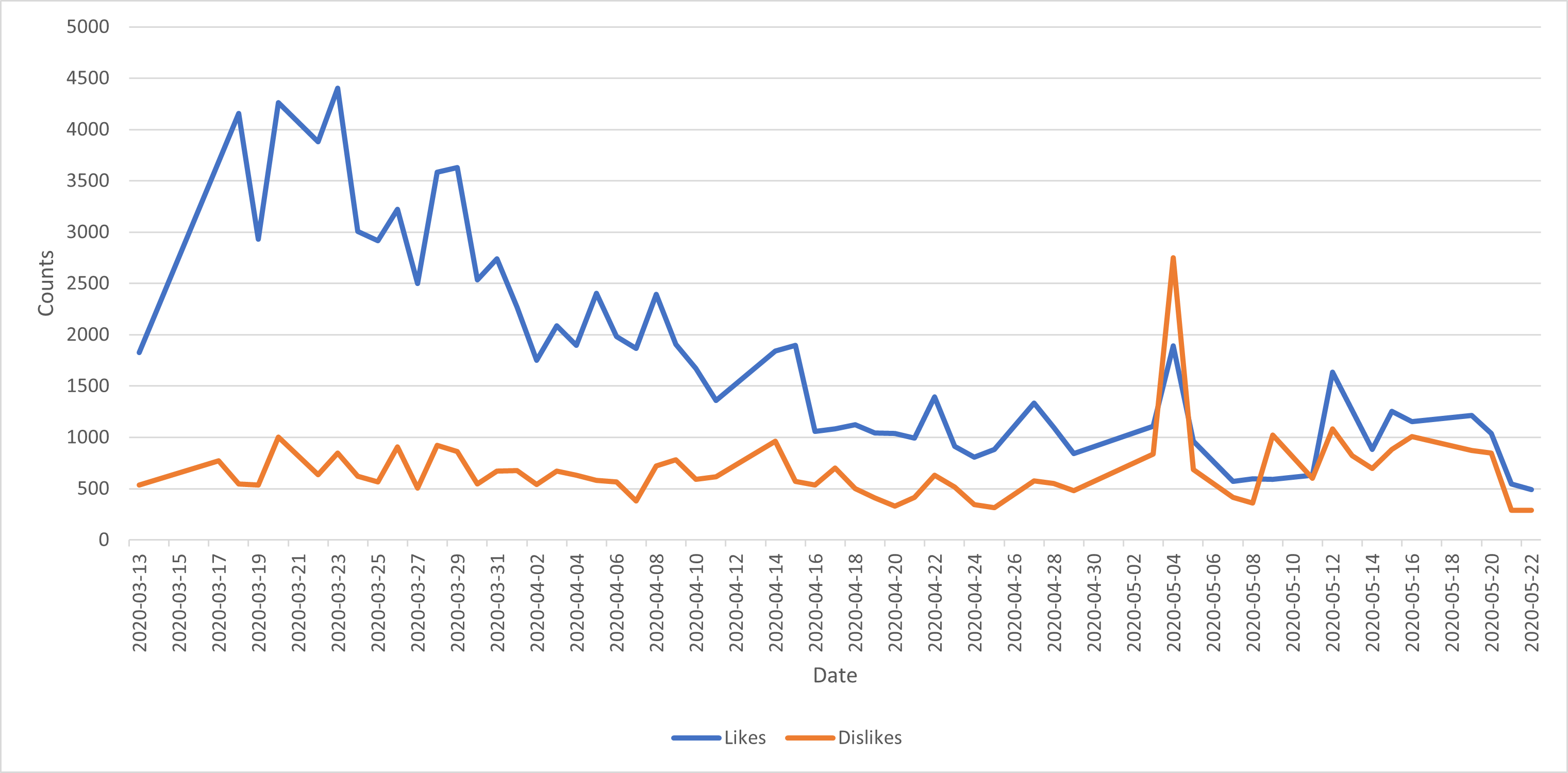

Supplement: Multimedia Appendix 3 [file jmir_v23i2e23957_app3.png]
